# Experiences of parents whose children participated in a longitudinal follow‐up study

**DOI:** 10.1111/hex.13473

**Published:** 2022-04-08

**Authors:** Nike Franke, Jennifer Rogers, Trecia Wouldes, Kim Ward, Gavin Brown, Monique Jonas, Peter Keegan, Jane Harding

**Affiliations:** ^1^ Liggins Institute, Faculty of Medical and Health Sciences University of Auckland Auckland New Zealand; ^2^ School of Medicine, Faculty of Medical and Health Sciences University of Auckland Auckland New Zealand; ^3^ Nursing, Faculty of Medical and Health Sciences University of Auckland Auckland New Zealand; ^4^ Learning, Development and Professional Practice, Faculty of Education and Social Work University of Auckland Auckland New Zealand; ^5^ Population Health, Faculty of Medical and Health Sciences University of Auckland Auckland New Zealand; ^6^ Te Puna Wananga, Faculty of Education and Social Work University of Auckland Auckland New Zealand

**Keywords:** consumer engagement, enabling factors, indigenous, long‐term research, paediatric trial, participant experience, research interests

## Abstract

**Background:**

Long‐term follow‐up is necessary to understand the impact of perinatal interventions. Exploring parents' motives and experiences in consenting to their children taking part in longitudinal studies and understanding what outcomes are important to families may enhance participation and mitigate the loss to follow‐up. As existing evidence is largely based on investigators' perspectives using Western samples, the present pilot study explored parents' perspectives in a multicultural New Zealand context.

**Methods:**

Data were generated using semi‐structured interviews with parents whose children had participated in a longitudinal study after neonatal recruitment. Parents' experiences of being part of the study were analysed thematically using an inductive approach.

**Results:**

Parents (*n* = 16) were generally happy with the outcomes measured. Additionally, parents were interested in lifelong goals such as the impact of parental diabetes. We identified three themes: (1) Facilitators: Research participation was aided by motives and parent and research characteristics such as wishing to help others and straightforward recruitment; (2) Barriers: A hesitancy to participate was due to technical and clinical research aspects, participation burden and cultural barriers, such as complex wording, time commitment and nonindigenous research and (3) Benefits: Children and parents experienced advantages such as the opportunity for education.

**Conclusions:**

Parents reported positive experiences and described the unexpected benefit of increasing families' health knowledge through participation. Improvements for current follow‐up studies were identified. Different ethnicities reported different experiences and perspectives, which warrants ongoing research, particularly with indigenous research participants.

**Patient or Public Contribution:**

No active partnership with parents of patients took place.

## 1 INTRODUCTION

Long‐term follow‐up is necessary to understand the impact of perinatal interventions but can be a challenge both in terms of recruitment and retention. Systematic reviews of retention strategies in longitudinal cohort studies revealed that reminder letters, resending questionnaires and offering incentives were the most successful specific tactics,^1^ while decreasing the burden on participants was the most effective general approach.^2^ However, studies investigating retention strategies are based on investigators' perspectives, rather than on those of research participants themselves. Understanding parents' motives and experiences in consenting to their children taking part in longitudinal studies and understanding what outcomes are important to families may enhance participation and mitigate the loss to follow‐up. Existing evidence generally lacks participants' perspectives, neglects long‐term perinatal trials and is mainly based on Western research. This pilot study addresses this gap by examining experiences and perspectives of an ethnically diverse sample of New Zealand parents who participated in a longitudinal perinatal trial and aims to identify research questions for a survey involving a large number of participants of follow‐up studies.

## 2 METHODS

### 2.1 Design

This qualitative study used a thematic analysis approach^3^ and adhered to the consolidated criteria framework (COREQ).^4^ Ethical approval was obtained from the University of Auckland Human Participants Ethics Committee (Ref. 024048).

### 2.2 Recruitment

Participants were parents of children who took part in the Children with Hypoglycaemia and their Later Development (CHYLD) study; a prospective cohort study investigating outcomes of children born at risk of neonatal hypoglycaemia. Children in the CHYLD study were assessed at birth, at 2, 4.5 and 9–10 years.^5^, ^6^, ^7^ Assessments included measures of growth, vision, brain structure (magnetic resonance imaging [MRI]), cognitive and language development, executive function, academic achievement and psychosocial adaptation. After each wave of data collection parents received a summary of the assessment findings. If the health or developmental assessments indicated children needed further assessments, a letter of referral was sent to their general practitioner with parental consent. A summary of the overall study findings at 2 and at 4.5 years was also sent to participating families at the time these were published. No other interaction between the research team and parents took place between the assessments. A purposive sample of parents whose children participated in the CHYLD study was invited to take part after their children had completed the 9–10‐year assessment. Sampling aimed to ensure the inclusion of a range of ethnicities with a specific focus on Māori, the indigenous population of New Zealand. Potential participants were approached by email, phone or in person.

### 2.3 Data collection

Participation options for the semi‐structured interview included in‐person, online face‐to‐face or via phone, either individually or in a group setting (i.e., focus group discussion). We invited parents to share their views about being part of the CHYLD study, focusing on what they liked and did not like, how being part of the study had affected them and which factors they perceived to be facilitators or barriers to participation. This discussion was guided by a list of topics, which was developed by the authors. Individual interviews were conducted by N. F. and J. R. (no parents opted for group discussion) between March and July 2020 and took between 15 and 45 min. Some data collection took place during the COVID‐19 lockdown in New Zealand and all public health precautions were followed. Respecting the indigenous value of whanaungatanga (kinship), interviews with Māori parents were conducted by J. R., who is of Māori descent (Ngāi Tahu) and who has been involved in recruiting these families to previous data collection waves. All other interviews were facilitated by N. F., who has a background in family psychology, and both N. F. and J. R. had prior experience in conducting and analysing interview data. Interviews were audio‐recorded and transcribed verbatim and checked for accuracy by N. F. No notes were taken during the interviews to encourage a natural conversation.

### 2.4 Data analysis

Qualitative data were analysed thematically to generate an authentic account reflecting parents' experiences with longitudinal perinatal research and to identify factors that may influence participation. The first step comprised reading the transcripts thoroughly and repeatedly, in line with an inductive approach.^3^, ^8^ The content of the data directed coding and theme development. Initial coding was followed by a grouping of the data, using QRS NVivo, to enable the identification of common themes. N. F. and J. R. reviewed and discussed data with all other authors to clarify and refine themes as analysis progressed and reached consensus regarding relevant extracts for each theme. The resulting themes and subthemes were refined and supported by quotations from parents' transcripts.

### 2.5 Validity and reliability

We took several steps to ensure the validity and reliability of the study findings. First, purposive sampling was employed to ensure a range of ethnicities to enhance the transferability of the results. Second, J. R. conducted the interviews with the Māori participants, with whom she had a previous research relationship. Third, regular discussions amongst authors were held while interpreting the results to minimize researcher bias. Fourth, the inductive approach to analysing the data involves applying a set of systematic procedures, which support the validity and reliability of the study.^8^ Fifth, we used quotations to support our findings, thereby allowing readers to review interpretation.

## 3 RESULTS

In total, 15 mothers and one father took part in the semi‐structured interviews. There were seven Pākehā (European descent) families, five Māori, three Indian and one Pacific Island family from a range of socioeconomic backgrounds (deciles 2–10 of the 2013 New Zealand Index of Deprivation).^9^ Participating children were aged between 9 and 13 years. See Table 1 for an overview of participants' demographic characteristics. Further details of recruitment rate and reasons for nonparticipation are reported in the COREQ checklist (Appendix A). All parents opted for an individual interview about their experience of participating in perinatal research, which mostly took place via video calling (*n* = 8), followed by phone (*n* = 4) and in‐person (*n* = 4). Parents were also asked which child health and wellbeing outcomes they were interested in learning more about, in addition to those tracked by the CHYLD study. Thematic analysis yielded three themes involving facilitators, barriers and benefits of research participation (Table 2).

**Table 1:** Demographic characteristics of the sample (*N* = 16)

ID	Parent	Ethnicity	NZDep 2013^a^ (1–10)	Residence	Sex of participating child	Age of participating child (years)
1	Mother	Māori	2	Urban	Female	13
2	Mother	Scottish	5	Rural	Male	13
3	Mother	NZ European	8	Urban	Male	12
4	Mother	Māori	10	Rural	Male	11
5	Mother	NZ European	6	Rural	Female	11
6	Mother	Samoan/NZ European	6	Urban	Male	10
7	Mother	Welsh/Zimbabwean	Unknown	Urban	Female	10
8	Mother	NZ European	2	Rural	Male	10
9	Mother	Indian	Unknown	Urban	Male	10
10	Mother	Māori	10	Rural	Male	10
11	Mother	Indian	Unknown	Urban	Female	10
12	Mother	Māori	9	Rural	Male twins	10
13	Mother	South African	7	Rural	Female	10
14	Mother	Indian	6	Urban	Male	10
15	Mother	Māori	Unknown	Rural	Male	10
16	Father	NZ European	5	Urban	Female	9

^a^ New Zealand Index of Deprivation 2013: Area‐based measure of socioeconomic deprivation in New Zealand displayed in deciles (1 = least deprived, 10 = most deprived).

**Table 2:** Overview of themes, subthemes and sub–subthemes

Theme	Subtheme	Sub–subtheme	Number of participants	Times mentioned
Facilitators	Motives	Help others and future generations	11	22
		Contribute to knowledge	7	9
		Personal education	5	6
		Value of research in general	5	5
		Contribute to clinical practice	1	3
		Avoid admission to NICU	1	1
		Represent ancestors	1	1
		Tailored feedback on child's health	1	1
		Value of CHYLD study	1	1
	Study characteristics	Recommends study	13	26
		Recruitment on site	9	11
		Flexibility in appointments	3	3
		Novel and interesting	3	5
		Koha (donation)	2	2
		Good study information	2	2
	Characteristics staff	Respect and care shown	9	12
		Continuous engagement	4	8
		Careful communication, e.g., reminders	4	4
		Shared power	3	7
		Feeling valued	3	3
		Familiar research staff	2	7
		Culturally responsive	2	4
		Professional and personable manner	1	1
	Parental characteristics	Actively looking for information	2	2
		Discuss potential participation	1	2
		High level of education	1	1
Barriers	Research‐related	Inadequate reports	7	23
		No informed consent	6	16
		Stressful blood tests	3	4
		Child did not understand the instructions	2	3
		Inexperienced research staff	2	3
		Inappropriate disclosure incidental findings	1	3
		Blind glucose monitoring	1	2
		Complicated study information	1	2
		Lengthy assessments	1	2
		Nonnatural intervention for baby	1	1
	Burden	Demanding research participation	7	17
		Time commitment	6	10
		Anxiety around enrolment first‐born	3	5
		Embarrassment re diabetes	3	4
		Worries re prolonged hospital stay	2	2
		Traumatic start in life	1	1
	Cultural	Nonindigenous research unsafe	1	4
		Difficulty articulating indigenous beliefs	1	1
Benefits	Children	Reports informative and useful	10	20
		Child happily involved	6	11
		Appreciative of gifts	3	4
		Regular breaks during assessments	1	1
		Testing in te reo (Māori language)	1	1
	Parents	Increased understanding child/family	9	22
		Reassurance of reports	4	13
		Reciprocity	3	3
		Access to support services	2	4
		Opportunity to learn	2	2

Abbreviations: CHYLD, Children with Hypoglycaemia and their Later Development; NICU, neonatal intensive care unit.

### 3.1 Overview of outcomes of interest

Parents described being satisfied with the information that they received about the outcomes measured, and identified guidance about referral for specialist care as of value. However, some parents were interested in tailored feedback on their child and their specific health status, as well as detailed comparisons to peers. Also, a strong focus for parents centred on the impact of each child's outcomes on lifelong goals, such as career paths, psychosocial development and physiological sequelae related to diet and parental diabetes. Parents wanted to know what the unique circumstances related to the study would mean for their child's developmental and academic strengths and weaknesses. Participant 7 described wishing to address areas of weakness and having awareness of strengths in terms of future career choices. She commented,

So, that's all relative to how you're going to turn out, what career choices and stuff you're going to take. So, if we can understand where he is with that and point him in the right direction and work on things that he perhaps is lacking at.

Participant 10 recommended that the study findings could be used to educate the wider community: ‘Continue the education, spread it far and wide. Because I know that sugar is a silent killer. And eating healthy, everything you put into your body, it's important’.

### 3.2 Theme 1: Facilitating factors

#### 3.2.1 Motives for research participation

Overwhelmingly, parents described elements of altruism by accepting the invitation to participate in research. Helping others and future generations seemed part of these parents' philosophical stance in life. Participant 5 commented that for her family taking part was a ‘no brainer’, as they wanted to ‘benefit future generations’. Participant 8 reported a specific interest in advancing clinical practice: ‘It would be interesting seeing if practice has changed’. The value of research, in general, was also referred to along with the wish to support the CHYLD study specifically. The most common personal reason for taking part in the CHYLD study was increasing parents' understanding of the current impact of diabetes on their own child or future grandchildren, with parents viewing research participation as an opportunity to educate themselves. One parent felt compelled to take part due to her role in the intergenerational link between her ancestors and future grandchildren. Other reasons for participating in the CHYLD study included the potential to prevent their child from being admitted to the neonatal intensive care unit and receiving tailored feedback on their child and their health status.

#### 3.2.2 Study characteristics

The recruitment process was described as straightforward and in some instances assisted with passing time waiting in hospital. For instance, Participant 13 reported: ‘I was just sitting bored in the hospital and got approached’. The decision to participate was made easy by the quality of the information provided, flexible scheduling of appointments, along the study process and follow‐up, which included the possibility of home visits. Most parents reported that taking part in the study was easy and that they found it interesting, describing the study as, ‘straightforward, interesting and enjoyable’. Parents would recommend study participation to other parents, saying it was not ‘onerous, costly or scary’. Parents were grateful for the koha (donation) that was provided by means of reimbursement, which for Participant 15 enabled research participation: ‘I remember just being so thankful that, I think I got a petrol voucher or something […] for us at the time, that meant I could take part’.

#### 3.2.3 Characteristics research team

An engaged, accommodating, respectful and culturally responsive research team was important to parents. Parents reported feeling connected with the study as the research team kept in touch with all parents throughout the years of the study, facilitating continuous engagement. Building relationships through respect and care over time by the research team was viewed as valuable. Indeed, the respect, care and compassion displayed by the research staff were acknowledged by most parents. Research etiquette, such as careful communication, reminding parents of an upcoming appointment and the professional and caring manner of research staff, was praised by parents. Participant 15 stated,

You always delivered [information] with care and compassion […] never have made me feel that I couldn't say yes or no […] I was never pushed, it was never something that I felt I had to be part of or anything like that…

Participant 2 recalled appreciating the professional and personable manner of a researcher who came on a home visit to disclose an incidental finding face‐to‐face. Parents also reported feeling valued and acknowledged. For example, two families appreciated being included in the clinical decision‐making and also in the care of their child. Moreover, strong connections were fostered by the presence of familiar research staff and the appropriate use of Māori values and customs. In contrast to data presented in Theme 2, one parent's willingness to overcome distrust of a Western, scientific worldview was facilitated by the openness of the research team who attended to this family's cultural wishes.

#### 3.2.4 Parental characteristics

A high level of education and the opportunity to discuss what participation entailed with a family member whose child was also enroled were personal facilitators. Two parents talked about how they actively looked for materials, such as videos on MRI, to help them make decisions about joining the study. As Participant 15 noted,

I analyse a lot of stuff and read a lot of stuff and make choices for my children based on the knowledge I've got. So, it's the knowledge I find about stuff […] I don't just blindly listen to what I'm told [laughing]. So, if I query it, then I'll go and I task myself with finding out why or, I'm making myself feel happy with a decision that I'm making.

### 3.3 Theme 2: Barriers to participation

#### 3.3.1 Technical and clinical research aspects

Aspects of the CHYLD study perceived as negative included difficulty in understanding the research, the effort required to read study materials, the need to consent to perceived nonnatural treatments for baby and frustration at not knowing glucose levels due to the use of a continuous monitor that stored data and was only downloaded at the end of the study. Four parents mistook the need to take blood samples as part of the study protocol rather than the hospital's protocol for routine monitoring of babies at risk of developing neonatal hypoglycaemia.

In one instance, the disclosure of a serious incidental finding by phone was considered inappropriate. Some parents described reports of the research findings as too complicated: ‘Umm, it was quite hard for me to actually understand it really […] I didn't really get what I was reading’ (Participant 12). Reports were viewed as too general, lacking appealing visuals such as graphs and not being child‐focussed. In regard to interactions with research staff, some parents reported that their child did not understand instructions, they were too shy to ask for further explanation, or that assessments were too long. Participant 16 also described the perception that the research staff was inexperienced with children with limited child‐focussed interactions: ‘And I think just some of them seeming nervous the way they talked to [my kids] before the assessments. Not a bad thing, it was just that you could tell they never had much to do with kids’. Another parent noted their reports were incorrectly named and one parent thought their child's test results did not reflect the child's ability.

#### 3.3.2 Burden of participation

In the context of the stress involved with dealing with the potential health issues of their infant, parents recounted their worries about whether study participation would lead to a prolonged hospital stay. Participation in longitudinal research was viewed as demanding and the additional challenge of enroling a firstborn child as anxiety‐provoking: ‘And like when it's your first baby, that's something that you're not prepared for’ (Participant 14). Deciding whether to participate was seen as particularly difficult when a baby had a traumatic start in life. Parents reported difficulties where both parents worked, were shift workers or were struggling with work–life balance: ‘My biggest thing was just trying to get time off work to sit in the appointment at one point in time, but that was just a work‐life balance thing’ (Participant 13). One parent felt embarrassed about having diabetes, stating that this prevented her from engaging with research staff.

#### 3.3.3 Cultural barriers

Māori parents described difficulty articulating indigenous beliefs around spirituality and the importance of avoiding transgression into tapu (sacred, restricted, supernatural condition): ‘Make sure you're safe and doing what you need to be doing’ (Participant 12). Reports were described as unresponsive to Māori world views. Participant 10 spoke about the challenge of overcoming her distrust in research based on non‐Māori values and the role of research staff in needing to make more effort attending to the needs of indigenous people to enable participation in research:

In the Māori‐diverse world, in our world, we are safe. Everything that we do, our practices and our techniques, keeps us safe. Therefore, allowing any European perspective in is not safe. So, you have to be courageous to take a chance on science. Because it is not our world, it doesn't belong to us and it's not in our genealogy.

### 3.4 Theme 3: Benefits of participation

#### 3.4.1 Benefits for children

Parents reported that their children were happily involved in the CHYLD study, enjoying tests and feeling special, especially on receiving a book after the 9–10 years assessment and a copy of the MRI scan, or appreciating doing something new. Participant 6 stated, ‘it is a really nice time for your own child to spend with them too. It's a special time for them to have’. Parents praised the presence of regular breaks during long assessments, the rigour of assessments and the use of te reo Māori (Māori language) in tests for Māori participants. Reports and assessment recordings were well received and seen as important in assisting with support for their child at school, or for the child to remember participation as they grew up. Although some found the reports too complicated, others viewed them as readable and informative: ‘it was a short printout of what they found: their results of how they were in the assessments, and where they sat on the scale, and how they expected or where they expected them to be’ (Participant 16).

#### 3.4.2 Benefits for parents

Parents considered the regular assessments reassuring, as described by Participant 13: ‘it was quite reassuring to kind of have other people looking at the development phases and just knowing that things were on track’. Parents noted how being part of the CHYLD study stimulated their interest in learning more about their child's health. Some parents viewed research participation as an opportunity to educate themselves. Research participation and the dissemination of the findings enabled an increase in parents' understanding of their child or their family dynamics. For example, Participant 9 said that being present during the assessment allowed them to see how their son interacted with strangers in a foreign language: ‘because at home, we can only see how the conversation is with our language […] the way he talks with outside people, that he's quite frank and confident’. Reciprocity of positive gains from research participation stood out, with parents reporting that they gained access to support services (e.g., parenting) or that they felt enriched by the experience, as described by Participant 10:

Youse (sic) have created this awareness, which has helped me to be proactive about it. And if youse weren't in our lives, I don't think we would be where we are. You know, we wouldn't have been as evolved as a whānau (family), myself and my children.

## 4 DISCUSSION

Overall, parents were very positive in recounting their experience of participating in a longitudinal study and most parents would recommend taking part in the CHYLD study to other parents. Parents expressed an interest in receiving information about the outcomes measured by the CHYLD study and also broader outcomes associated with lifelong goals, such as children's academic strengths and weaknesses, career paths and the impact of parental diabetes on children's physiology. Parental participation in the CHYLD study was affected by facilitating factors, barriers to participation and benefits of participation.

The research team was generally praised for their cultural sensitivity, which is an important facilitator for Māori.^10^ Other facilitators for ongoing participation were reminders of a follow‐up assessment, flexibility of scheduling and assessment location (i.e., home visits), which were the most frequently used retainment strategies identified in a systematic review of retention strategies.^2^ Continuous engagement, cultivation of connections and the compassion and care shown by the research team were additional facilitating factors that were not reported in the systematic review. These facilitators in our sample population may be reflective of the importance of the role of human relationships within Māori/Polynesian cultures in New Zealand^11^ and confirm some of the retention strategies adopted by a large New Zealand prebirth cohort study, such as engagement through birthday cards and newsletters and the careful selection of interviewers.^12^

Authors of a birth cohort study recommended the inclusion of altruism as a recruitment focus, especially in studies with few perceived benefits.^13^ Ostensible altruistic motives found in our study (i.e., helping others), may have originated from parents' desire to reciprocate for care that has benefitted their child. Indeed, our findings around the importance of engagement and connections imply that reciprocity is of great significance to families. Therefore, it may be more helpful to promote a study's social and altruistic benefits.^14^ One parent's wish to use CHYLD research findings for education purposes further corroborates the importance of social benefits. Personal motivators found in the current study included the desire to serve the interests of other family members and the opportunity to learn, but monetary compensation was seen as an unexpected benefit, contrasting with a prior study where financial gains were a major motivator.^15^

Informed consent and awareness of the study's objectives were variable and four parents were unaware that blood tests were standard hospital procedure. As stated by policies that govern research with human participants, the consent process should focus on comprehension of what study participation entails, especially when studies intersect with clinical care.^16^ Detailed consent forms that promote participants' interests and rights appear to be aimed at protecting the research institution^17^ rather than increasing participants' understanding.^18^ Indeed, lengthy and technical study information sheets were mentioned as a barrier to informed consent, suggesting that complex study information is not conducive to autonomous informed consent.^16^ A potentially effective strategy to reach informed consent is to provide prospective participants with reasons for and against study participation,^19^ an approach employed by one parent in the pilot study who discussed what participation entailed with a family member whose child was also enroled. Familiarity with research staff may also promote discussions around participation that enable participants to decide whether the research aligns with their interests and values.^20^

Common barriers to research participation included parental burden, with parents struggling to find time to commit, confirming prior research.^13^, ^15^ In line with the general retention strategy of decreasing the burden of participation presented in a systematic review, onsite recruitment minimizes the strain on prospective participants' time.^2^ However, if onsite recruitment takes place around childbirth, this could hinder informed consent through a lack of opportunity to discuss participation and look for additional information. The stress involved with having a baby with potential health concerns may also impede informed consent, especially with first‐time parents. A cultural barrier for Māori parents was their distrust in research based on nonindigenous values, which required courage to overcome. When Western, individualistic medical research is conducted in non‐Western settings, participants may experience distrust as medical decisions usually involve consultation with leaders in the community or family.^21^ This is of significance in New Zealand, where over a third of the population comprises ethnicities originating from cultures that place more emphasis on connections to the community or family rather than the individual.^22^ Therefore, informed consent should take into account participants' cultural background, including having research staff of the same ethnic group as the participant,^23^ and may require seeking community‐level informed consent before obtaining individual consent.^24^ Māori voice may be promoted by encouraging relevance to Māori, such as meaningful reporting of the findings, and by inspiring research staff to familiarize themselves with concepts of Māori ethnicity, ancestry and descent.^10^

Māori parents appreciated that child assessments were offered in te reo Māori. This choice in assessment language acknowledges the standing of te reo Māori, shows respect for Māori entitlements to speak te reo Māori and acknowledges the standing of Māori as sovereign partners under New Zealand's founding document, te Tiriti o Waitangi (The Treaty of Waitangi).^25^ Other perceived benefits included access to support services, reassurance offered by regular assessments and the unexpected advantage of increased health knowledge. These benefits, including receiving reports and other study materials, emphasize the reciprocity of research participation and may elicit a sense of gratitude and obligation to remain in the study.^26^

A limitation of the current pilot study was the inability to engage with each participant in person. Due to the restrictions of COVID‐19, most interviews were held remotely (i.e., via the internet or phone). For Māori/Polynesian cultures in‐person contact is respectful and accepted practice. In addition, internet and mobile phone networks in New Zealand do not reach all rural areas. These factors may have led to the involuntary exclusion of some Māori/Polynesian families and those living in remote areas. Also, these findings were based on 16 participants and need to be corroborated in a larger sample. Since saturation was not reached, nor intended, important experiences may potentially be undetected. Lastly, parents were not involved in the development of the topic guide as a common goal of pilot studies is to obtain participants' comments on interview questions to inform a subsequent study.^27^ To avoid misinterpretation of any questions during the interviews, participants were encouraged to ask for clarification or to expand on questions they felt unsure about.

## 5 CONCLUSIONS

This is one of few studies investigating the perspectives of parents on outcomes of importance to families and their views about what enables and deters participation in longitudinal research studies. The parental views reported in this pilot study offer insight into how longitudinal perinatal studies can be designed and executed to increase retention and enhance the benefits of participation to families. Potential recruitment foci include benefits to parents' children or future grandchildren and the opportunity to educate those involved and the wider community. Retention may be enhanced by offering flexible scheduling and home visits and by creating reciprocal relationships. An increase in research uptake by Māori may be achieved by ensuring that all research staff are familiar with and able to participate in Māori customs and values and by approaching community leaders before obtaining individual consent. Current findings, including the different experiences and views by participants of different ethnicities, warrant ongoing research, particularly with Māori/Polynesian participants.

## CONFLICTS OF INTEREST

The authors declare that there are no conflicts of interest.

## AUTHOR CONTRIBUTIONS

*Nike Franke*: Data collection, transcribing interviews, data analysis, drafting manuscript, revision manuscript. *Jennifer Rogers*: Design and conceptualization study, data collection, data analysis, revision manuscript. *Trecia Wouldes*: Design and conceptualization study, advisor data analysis, revision manuscript. *Kim Ward*: Design and conceptualization study, advisor data analysis, revision manuscript. *Gavin Brown*: Design and conceptualization study, advisor data‐analysis, revision manuscript. *Monique Jonas*: Design and conceptualization study, revision manuscript. *Peter Keegan*: Design and conceptualization study, advisor data analysis, revision manuscript. *Jane Harding*: Design and conceptualization study, revision manuscript.

## Data Availability

Data were not be shared due to privacy/ethical restrictions. The data that support the findings of this study are not shared due to privacy or ethical restrictions.

## APPENDIX A

CONSOLIDATED CRITERIA FOR REPORTING QUALITATIVE STUDIES (COREQ): 32‐ITEM CHECKLIST

This checklist is intended to supplement the manuscript by providing further detail on methodology.

Domain 1: Research team and reflexivity

Personal characteristics

1. Interviewers: Nike Franke and Jennifer Rogers

2. Credentials and 3. Occupation:

N. F. (PhD)—Research fellow, Liggins Institute, University of Auckland

J. R. (MHS)—Follow‐up and Māori engagement lead, Liggins Institute, University of Auckland

4. Gender and ethnicity:

N. F.—Female, New Zealand European

J. R.—Female, Māori (Ngāi Tahu)

5. Experience and training: Both N. F. and J. R. have conducted and analysed semi‐structured interviews before the current pilot study.

*Relationship with participants*

6. Relationship established:

N. F.: Had no established relationship with any of the participants

J. R.: Had established relationships with her participants through her long‐term involvement with assessments and contact tracing within the Children with Hypoglycaemia and their Later Development (CHYLD) study

7. Participant knowledge of the interviewer:

Participants were aware of the reasons for conducting the pilot study, which were detailed in the participant information sheet and consent form. Both J. R. and N. F. outlined their role in the pilot study before obtaining consent. N. F. was new to the CHYLD study research team. J. R. had long‐term relationships with the participants she interviewed as part of her activities related to the CHYLD study.

8. Interviewer characteristics:

J. R. is a Māori researcher and N. F. is of European descent. This mixed indigenous—nonindigenous partnership assisted with establishing rapport with participants, depending on their background. Also assisting with rapport and with the interpretation of the data was both researchers being parents/grandparents.

Domain 2: Study design

*Theoretical framework*

9. Methodological orientation and theory: Thematic analysis

*Participant selection*

10. Sampling: We used purposive sampling to ensure a spread of ethnicity and to ensure representation of Māori, the indigenous population of New Zealand.

11. Method of approach:

Most participants were sent an email containing the participant information sheet and consent form. This was followed up by a phone call. Some participants of Māori descent were approached in person instead. The current study took place during the COVID‐19 pandemic when New Zealand was in lockdown for an extended period of time.

12. Sample size: *N* = 16

13. Nonparticipation:

We approached 72 participants of the CHYLD study. A total of 25 did not respond to any form of contact; 15 declined, due to being busy; 12 agreed, but could not be further contacted; 4 agreed, only for in‐person group discussion which was impossible during COVID‐19.

*Setting*

14. Setting of data collection: All participants were at their homes or workplace.

15. Presence of nonparticipants:

Typically, only the participant and interviewer were present. In some instances, other family members of the participant, such as a partner or young child were also present.

16. Description of sample:

There was one father and 15 mothers. Ethnicities included Māori (*n* = 5), NZ European (*n* = 4), Indian (*n* = 3), Samoan/NZ European (*n* = 1), Welsh/Zimbabwean (*n* = 1), South African (*n* = 1), Scottish (*n* = 1). Families from eight urban and eight rural locations were included and the age of the children at the time of data collection ranged from 9 to 13 years.

*Data collection*

17. Interview guide:

All interviews were guided by a set of questions and prompts. During in‐person interviews, participants were given a copy of the questions and prompts. The current project is a pilot project and will inform the next phase.

18. Repeat interviews: No repeat interviews were carried out.

19. Audio/visual recording: Interviews were audio‐recorded and transcribed ad verbum by NF.

20. Field notes: No notes were made during the interviews.

21. Duration: The interviews took between 15 and 45 min.

22. Data saturation: Saturation was not reached, nor was saturation the goal of this pilot study.

23. Transcripts returned: Transcripts were not returned to participants for correction.

Domain 3: Analysis and findings

*Data analysis*

24. Number of data coders:

There were two coders (N. F. and J. R.) and four advisors (T. W., K. W., P. K. and G. B.). After the coding matrix was constructed and a consensus was reached, all transcripts were coded again by N. F.

25. Description of the coding tree: A table with major and minor themes is presented in the manuscript, together with a description in the text.

26. Derivation of themes: All themes were derived directly from the data.

27. Software: QRS NVivo was used to manage the data.

28. Participant checking: Participants did not provide feedback on the findings.

*Reporting*

29. Quotations presented: Participant quotations are presented to illustrate the findings, identified by a study number.

30. Data and findings consistent: There was consistency between the data presented and the findings.

31. and 32. Clarity of major and minor themes:

A distinction was made between major and minor themes. Subthemes were presented to illustrate differences between participants' experiences within each major theme.
